# Meetings of Societies

**Published:** 1931

**Authors:** 


### Meetings of Societies

### Bristol Medcio-Chirurgical Society

SESSION 1930-31.

THE Annual General Meeting of the Society was held in the Physiological Lecture Theatre of the University on Wednesday, 14th October, 1931, at 8.30 p.m.

Professor D. C. Rayner was in the Chair, and ninety-two members were present.

The minutes of the previous meeting were read and confirmed.

Three ladies and five gentlemen were proposed for election to the membership of the Society.

The President, Professor D. C. Rayner, then resigned the Chair to his successor, Dr. J. R. Charles.

### BRISTOL MEDICO-CHIRURGICAL SOCIETY.

### INCOME AND EXPENDITURE ACCOUNT for the year ended 30th September, 1931.

### INCOME.

| | £ | s. | d. | £ | s. | d. |
|---|---|---|---|---|---|---|
| Members' Subscriptions .. | 300 | 15 | 6 | | | |
| Do., outstanding for 1930–1931 .. .. .. .. | 55 | 2 | 6 | | | |
| | | | | 355 | 18 | 0 |
| Interest on Bank Deposits .. .. | | | | 3 | 16 | 9 |
| Interest on £500 4 per cent. Consolidated Stock, less tax .. .. | | | | 15 | 10 | 0 |
| | | | | £375 | 4 | 9 |

### EXPENDITURE.

| | £ | s. | d. | £ | s. | d. | £ | s. | d. |
|---|---|---|---|---|---|---|---|---|---|
| Grant to *The Bristol Medico-Chirurgical Journal* .. .. .. | | | | | | | 275 | 18 | 11 |
| Librarian—Honorarium .. .. .. | | | | | | | 15 | 0 | 0 |
| Miscellaneous Expenses— | | | | | | | | | |
| Goodway–Refreshments | | | | 22 | 10 | 0 | | | |
| Postages and Sundries | | | | 8 | 13 | 5 | | | |
| Printing and Stationery | | | | 11 | 7 | 3 | | | |
| Audit Fee .. .. .. .. | | | | 2 | 2 | 0 | | | |
| Lantern and Cinematograph at Meetings .. | | | | 4 | 4 | 0 | | | |
| University Marshal .. | | | | 1 | 0 | 0 | | | |
| Lewis's Medical and Scientific Library— | | | | | | | | | |
| Subscription | 4 | 4 | 0 | | | | | | |
| Postages .. | 0 | 8 | 7 | | | | | | |
| | | | | 4 | 12 | 7 | | | |
| | | | | | | | 54 | 9 | 3 |
| Balance, being excess of Income over Expenditure carried to Capital Account .. .. .. .. .. .. .. | | | | | | | 29 | 16 | 7 |
| | | | | | | | £375 | 4 | 9 |

### BALANCE SHEET as at 30th September, 1931.

### CAPITAL AND LIABILITIES.

| | £ | s. | d. | £ | s. | d. |
|---|---|---|---|---|---|---|
| Sundry Creditors .. .. | | | | 85 | 10 | 8 |
| Capital as at 30th Sept., 1930 .. .. .. .. .. | 656 | 8 | 11 | | | |
| *Add* Balance of Income and Expenditure Account as above .. | 29 | 16 | 7 | | | |
| | | | | 686 | 5 | 6 |
| | | | | £771 | 16 | 2 |

### ASSETS.

| | £ | s. | d. | £ | s. | d. |
|---|---|---|---|---|---|---|
| Cash at Bank— | | | | | | |
| Current Account .. .. | 114 | 7 | 8 | | | |
| Deposit Account .. .. | 157 | 7 | 0 | | | |
| | | | | 271 | 14 | 8 |
| £500 4 per cent. Consolidated Stock at cost .. .. .. .. .. .. .. .. | | | | 437 | 9 | 0 |
| Members' Subscriptions for 1930–1931 outstanding | 55 | 2 | 6 | | | |
| Roberts — Honorarium in advance for 1931–32 .. | 7 | 10 | 0 | | | |
| | | | | 62 | 12 | 6 |
| | | | | £771 | 16 | 2 |

Audited and found correct,

H. E. KEELER,

*Chartered Accountant,*

*Auditor.*

16–18 Clare Street, Bristol,

12*th October*, 1931.

Dr. Watson-Williams proposed a vote of thanks to Professor Rayner for his services during the past year ; this was seconded by Dr. Symes and carried with acclamation. Professor Rayner replied.

Dr. J. R. Charles then gave his inaugural address on **"Structure and Function."**

Mr. T. Carwardine proposed a vote of thanks to the President for his address ; this was seconded by Dr. Carleton and carried with acclamation.

The Hon. Secretary and Hon. Treasurer then gave their reports, and the Editor gave his report on the production and development of the *Journal.*

The President thanked them for their reports and for their work during the past year.

Professor Hey Groves was elected to the office of President-Elect, on the proposition of Professor Nixon, seconded by Dr. Kennedy.

Dr. Kyle proposed and Mr. N. Kemm seconded the election of Mr. H. L. Shepherd as Hon. Secretary. This was carried.

Mr. A. E. Iles was re-elected Hon. Treasurer on the proposal of Mr. Duncan Wood, seconded by Dr. R. C. Clarke.

Dr. Watson-Williams proposed that Dr. G. Parker be elected Hon. Medical Librarian ; this was seconded by Mr. A. E. Iles and carried.

The following gentlemen were elected to serve on the Committee, proposed by Dr. R. S. Statham and seconded by Dr. Ormerod :—

Dr. W. K. Wills.

Mr. H. Chitty.

Dr. H. G. Kyle.

Dr. Carey F. Coombs.

Mr. W. A. Jackman.

Mr. Duncan Wood.

Mr. A. R. Short.

Dr. W. P. Kennedy.

Dr. Elwin Harris (jun.).

Dr. G. Parker, Mr. A. R. Short and Dr. J. O. Symes were elected to serve on the University Sub-Committee for the Medical Library on the proposal of Dr. P. Phillips, seconded by Dr. E. G. Bradbeer.

The President then referred to the Annual Dinner, and suggested that, on account of the present national financial and political crisis, there should be no dinner this year. This was carried and, furthermore, that members should be asked to contribute to a fund, which should be forwarded as from the Society, half to the Medical Benevolent Fund and half to the Lord Mayor of Bristol's Relief Fund.

The second meeting of the session was held in the Physiological Lecture Theatre of the University on Wednesday, 11th November, 1931, at 8.30 p.m. Dr. J. R. CHARLES in the Chair.

The minutes of the previous meeting were read and confirmed.

Dr. A. D. FRASER showed a number of interesting pathological specimens.

Professor J. A. NIXON and Dr. G. B. BUSH read papers* on "**Silicosis,**" Dr. Bush illustrating his paper with a series of skiagrams and also with the epidiascope.

Dr. MAYES discussed the prognosis in cases of Silcosis, quoting a group of thirty in which only four were found to be living four years after his first examination.

Dr. CARLETON mentioned that catarrhal children also developed pulmonary fibrosis, but in them the condition leads on to bronchiectasis rather than tuberculosis. He inquired whether cases of silicosis also developed bronchiectasis or radial dilatation of the bronchioles. In his experience suction dyspnœa was common in fibrosis.

Dr. KENNEDY spoke of the effects of silica poisoning in the spleen, liver and stomach. He quoted a case of recovery from pulmonary silicosis.

Dr. ALEXANDER spoke of a patient, at present under his care, in whom silicosis was due to sand-blasting.

Dr. BERGIN congratulated Dr. Bush on the excellence of his skiagrams and stressed the value of radiology in the early diagnosis of silicosis.

The PRESIDENT said he considered that the expense to the workmen of examination by the Silicosis Board might act as a deterrent to justifiable claims, and spoke of the length of time the disease might exist before its presence was suspected.

Professor NIXON and Dr. BUSH replied.

---

The third meeting of the session was held in the Physiological Lecture Theatre of the University on Wednesday, 9th December, 1931, at 8.30 p.m. Dr. J. R. CHARLES in the Chair.

The minutes of the previous meeting were read and confirmed.

* See pages 253 and 259.

Dr. A. L. TAYLOR showed pathological specimens, and Dr. D. A. ALEXANDER showed a specimen of a lung from the case of silicosis which he had mentioned at the previous meeting.

Dr. R. G. GORDON and Dr. M. F. FORRESTER-BROWN showed a cinematograph film illustrating the **"Treatment of Anterior Poliomyelitis."**

A discussion on the film and its subject then followed.

---

We greatly regret that in the discussion on Dr. BRISTOWE'S paper on **"The Sterilization of the Unfit,"** as reported in our last issue, a mistake was made in the reporting of Dr. A. N. P. Milner's remarks. What he actually said was :—

"Of recent years the Neo-Darwin theory appears to have fallen into disrepute ; even Shaw describes it as Unnatural Selection, while evidence is accumulating in favour of the theory of Creative Evolution. It is probably unwise to prophesy, but it may be that in future we shall have to revise our present attitude to Evolution and Mendelism, and possibly we shall find that inheritance occurs, not by structural transmission, but by the transmission of functional diatheses."

